# Green and novel ultrasonic extraction with UHPLC-MSMS analysis of natural sweetener (Glycyrrhizic acid) from *Glycyrrhiza glabra*; a multifactorial mechanistic evaluation based on statistical analysis

**DOI:** 10.1016/j.ultsonch.2021.105696

**Published:** 2021-07-29

**Authors:** Rizwan Ahmad, Mohammed Aldholmi, Aljawharah Alqathama, Saeed Aldossary, Salem Bubshait, Muath Aljaber, Asma Abuhassan, Alaa Aldarwish, Leena Alateeq

**Affiliations:** a Department of Natural Products and Alternative Medicine, College of Clinical Pharmacy, Imam Abdulrahman Bin Faisal University, P.O. Box # 1982, Dammam 31441, Saudi Arabia; b Department of Pharmacognosy, Pharmacy College, Umm Al-Qura University, Makkah 21955, Saudi Arabia; c College of Clinical Pharmacy, Imam Abdulrahman Bin Faisal University, Dammam 31441, Saudi Arabia

**Keywords:** Glycyrrhizic acid, Ultrasonication, UHPLC-MSMS, Cavitation, Sonochemical, PCA

## Abstract

A novel, green and eco-friendly, cost-effective, fast, and reliable high energy ultrasonication (US) extraction with UHPLC-MSMS (Ultrahigh performance liquid chromatography with mass spectrometry) quantification of Glycyrrhizic acid (GZA) is reported herein for the first time. The study provides useful insights regarding the effect of US-factors with statistical analysis and mechanisms, involved in GZA-extraction and analysis. An US-extraction method (US-MD) was developed using three levels of US factors: solvents (AC (acetone), EtOH (ethanol), H_2_O (water)), time (1, 2, 3 min), amplitudes (30, 40, 50%), pulse (10/0.5, 20/0.5, 30/0.5 sec), particle sizes (0.5, 1, 1.4 mm), and temperatures (20, 30, 40 °C). The US-MD was further validated with high accuracy 98.96 ± 6.82 and r^2^ = 0.995 whereas, an in-house analytical method (UHPLC-MSMS) was developed and validated to quantify the GZAamount. UHPLCMSMS-MD resulted in a retention time of 0.31 min with MSMS (821.400 > 351.200) in a 1 min run time whereas, UHPLCMSMS-MV showed high accuracy and precision with r^2^ = 0.998 for GZA. Statistical analysis of K-mean clustering finalized US-set-of-factors showing optimum extract yield (mg/1mg) of 0.48 with sum (2.41 ± 014) and mean (0.27) along with a high GZA-amount (μg/mg) of 8.23 with sum (43.31 ± 2.07) and mean (4.81) for H_2_O in 3 min at 40 °C using particle size (1.4 mm), amplitude (50%), and pulse (30/0.5). Large scale application of US-UHPLCMSMS confirmed the evaluation power of the method showing the order for GZA amount; Egypt > Pakistan > Syria > India > Palestine > America > Georgia > Morocco. A significant effect for US factors Vs extract yield and GZA amount was observed however, solvent*GZA-amount and extract yield*particle size were more significantly correlated compared to time*temperature*amplitude*pulse analyzed via PCA, GLM-UniANOVA, K-mean, and Pearson’s correlation (*P ≤ 0.05*). A combined mechanism of shear stress, macroturbulence due to acoustic cavitation and implosions, sonochemical, and sonocapillary effect were noted for the US technique producing higher extract yield and GZA amount from licorice.

## Highlights

• Green and novel US-extraction and UHPLC-MSMS analysis method was developed/validated.
• Temperatures, solvents, amplitudes, pulse rates, and particle sizes were correlated with GZA.
• Cavitation, turbulence, sonocapillary, and sonochemical effects were observedduring US-process.
• A linear increase was observed with an increase in US-factors with an exception of particle size.
• Solvent showed more correlation with GZA whereas, particle size more towards extract yield.

## 1 Introduction

Licorice (*Glycyrrhiza glabra*) is one of the oldest plants with widespread applications in food, pharmaceutical products, and cosmetics. It is considered a perfect sweetening agent (50–100 times sweeter than sucrose) used to mask the bitter taste, hence used as an additive [1]. In medicine, its benefits have been reported in various diseases including anti-viral, anti-inflammatory, anti-microbial, anti-cancer, anti-diabetic, respiratory disorders, hyperdipsia, epilepsy, fever, sexual debility, paralysis, stomach ulcers, rheumatism, skin diseases, hemorrhagic diseases, and jaundice. Additionally, the antioxidant activity has been studied with significant outcomes in various health problems [2]. The emerging uses for licorice are witnessed by enormous skin care products where it plays a vital role as the main constituent. The market value and importance of licorice is rising on a regular basis [3], where a global value of 1700 M USD has been calculated for licorice extract. This value is expected to grow at a faster rate in successive years ahead [4]. In the global marketing system, licorice root costs an average price of 4.11 USD/Kg (subjected to variation in price). Currently, China is considered amongst the top licorice producers with a gross production volume of 132 and an import value of 63.02 thousand tons. With regard to global export, China tops the exporter list with a share of 25.88% (795.99 M USD) out of total global export value (3.08B USD) followed by the United States of America (value of 13.33%), Germany, India, Japan, South Korea, French, and, Taiwan [5]. Saudi Arabia (0.01% global import volume) imports around 177,000 USD licorice extract from Syria (59.1%), Turkey (27.7%), and Egypt (13.1%) as per 2018 report [6], [7]. This high market value for licorice is due to its unique phytochemistry with the presence of alkaloids, glycoside, carbohydrates, starch, phenolic compounds, flavonoids, proteins, pectin, mucilage, saponins, lipids, tannins, sterols, and steroids [8]. A number of extraction and quantification methods have been reported to extract the important phytochemicals from licorice including the natural sweetener Glycyrrhizic acid (GZA). These methods consisted of; Ultrasound and salt-assisted liquid–liquid extraction with an analysis time of 20 mins [9], MAE (microwave-assisted extraction) used ethanol–water mixture and ammonia in different concentrations with GZA retention time of 4.35 min [10], oven-dried licorice extracted with 50 mL ethanol–water at time duration of more than 1 hr. and quantified with HPLC using methanol and acetic acid in the mobile phase [1], an advance supercritical CO_2_ extraction with methanol in 40–120 min and quantification via HPLC using methanol and acetic acid in the mobile phase [11], and a green extraction using deep eutectic solvents in 30 min and an HPLC analysis with 15 min analysis time [12]. Mukhopadhyay & Panja, 2008 reported a summary of the GZA extraction and analysis methods where different processes of MAE, stirring and centrifugation, precipitation, sonication in an ultrasonic bath, and micro-Soxhlet have been mentioned [13]. However, the reported methods were unable to present a complete and unique set of extraction along with analysis with one or more of the loopholes during extraction and analysis including; the use of toxic solvents, lack of use of green solvents, complex mobile phases, less sensitive and reproducible methods using HPLC, and utilization of time- and solvent-consuming methods for extraction and analysis.

The current study aims to investigate the extraction and analysis of the natural sweetener, GZA, using a high energy Ultrasonication technique (20 kHz; 500 Watt) with an advanced and sensitive analytical technique of ultra-high pressure liquid chromatography attached with mass spectrometry (UHPLC/MSMS). Green and ecofriendly solvents are intended to be used throughout the extractions, samples preparation, and analysis processes. The unique feature for this study is the evaluation of all the available set of factors which may affect the yield and recovery of GZA during extraction and analysis. The US (high energy ultrasonication-technique) factors of time, pulse rate, amplitude, temperature, solvent, and particle size will be investigated herein. For analysis via UHPLC/MSMS, a simple mobile phase using green solvents (without any additives) and a fast and reproducible method will be developed and validated. Furthermore, the effects of the US factors will be explained with relevant mechanisms and various statistical methods will be applied to evaluate the significant multifactorial effect upon extraction and analysis of GZA using US-UHPLC/MSMS.

## 2 Experimental

### 2.1 Instrument used

US (high energy ultrasonication technique) consisting of 20-kHz (50 Watt) ultrasonic processor (Fisher Scientific, 2000 Park Lane Pittsburgh, PA, USA) attached to Transducer (Model CL-334), a fixed horn (220-A) and a removable Titanium probe (420-A; 1 mm diameter). The US processor was supplied with a display power supply. UHPLC/MSMS (ultra-high-pressure Liquid chromatography-mass spectrometry instrument i.e. LCMS-8050, Shimadzu, Japan) in-built with a tunable high-resolution triple-quadrupole-ESI (electrospray ionization source) mass detector of LCMS-8050, Shimadzu, Japan), instrument controller (CBM-20A), degassing unit (DGU-30A), auto-sampler (SIL30AC), binary pumps solvent system (LC-30AD), thermostatted column compartment (CTO30A) and a photodiode array detector (SPD-M20A). BUCHI Rotavapor® [R-215, Postfach, Flawil, Switzerland] and Thermo Scientific™ Reacti-Therm™ Heating and Stirring Modules (Reacti-therm III # TS-18824 Heating module & Reacti-Vap III# TS-18826 evaporation unit, Rockford, IL, USA) were used to evaporate and dry the solvents after extraction. Sieve/mesh; stainless steel sieve with different apertures i.e. 0.5, 1.0, and 1.40 mm (Laboratory test sieve, BS 410-1, Endecotts Ltd., London, England). The chromatographic column for analysis was a C-18 Cyano column (Pinnacle DB; 1.9 µm; 30 mm × 2.1 mm).

### 2.2 Chemicals and reagents used

Glycyrrhizic acid (GZA) from RxBiosciences (Bonanza way, Gaithersburg, MD20879, USA), analytical as well as LCMS grade ethanol (EtOH), and analytical grade acetone (ACE) were obtained from Merck (Darmstadt, Germany). For sample preparation, extraction, and UHPLC/MSMS mobile phase an in-house Pure Lab (ELGA, High Wycombe, UK) purified water (H_2_O) was used.

### 2.3 Samples used

In this study, eight different geographical origin licorice root samples (Syria, Egypt, America, Pakistan, India, Palestine, Georgia, and Morocco) were collected from the local markets at Khobar and Dammam, Kingdom of Saudi Arabia. The roots were properly cleaned, cut into small pieces, and ground to powder form. The powder was further sieved into three different sizes of 0.5–1.4 mm. One mg and 10 g of the samples were used respectively for method development (US-MD) and evaluation of large scale US-application in licorice samples from different geographical origins, respectively.

### 2.4 US MDMV (ultrasonication method development and validation)

#### 2.4.1 US-MD (ultrasonication method development)

An in-house method was developed where a set of different factors was evaluated in a proper range as mentioned: solvent (AC, EtOH, H_2_O), time (1, 2 and 3 min), amplitude (30, 40 and 50%), pulse (interval) (10/0.5, 20/0.5 and 30/0.5 sec), particle size (0.5, 1 and 1.4 mm), and temperature (20, 30 and 40 °C). Based on three levels of each factor (low to high) for each sample, a total of fifty-four different samples (1 mg each) were prepared, dissolved in 10 mL of the respective solvents and subjected to US extraction under a specified set of extraction in a sequential manner. The 54 samples were divided into two batches (27 samples in each batch) where one batch (1–27) was extracted at ambient temperature and the second batch (28–54) was extracted at different temperatures. The details for each sample along with the set of factors (solvent, time, amplitude, pulse, particle size, and temperature) used for US extraction are shown in Table 1.

**Table 1:** mean, sum, and range for extract yield and GZA-amount observed Vs different set of US-factors (samples 1–27 represents set of data for extraction without temperature; 28–54 represents extraction under a various set of temperatures).

Sample #	US factors values tested				Temperature (°C)	GZA amount (μg/mg) in different solvents			Extract yield in different solvents (mg/1mg)		
	Time (min)	Amplitude (%)	Pulse (seconds)	Particle size (mm)		AC	EtOH	H_2_O	AC	EtOH	H_2_O
1–27	1	30	10/0.5	0.5	0	0.50	0.80	2.08	0.03	0.02	0.06
	2	40	20/0.5			0.78	1.48	3.82	0.05	0.04	0.06
	3	50	30/0.5			0.80	1.78	5.59	0.11	0.04	0.12
	1	30	10/0.5	1		0.78	1.11	2.53	0.10	0.03	0.19
	2	40	20/0.5			0.80	1.72	4.86	0.27	0.05	0.26
	3	50	30/0.5			0.85	2.00	5.63	0.34	0.07	0.41
	1	30	10/0.5	1.4		0.10	1.55	2.70	0.24	0.05	0.29
	2	40	20/0.5			0.79	1.93	6.51	0.30	0.07	0.39
	3	50	30/0.5			0.87	2.54	7.56	0.38	0.15	0.43
28–54	1	30	10/0.5	0.5	20	1.33	1.35	2.56	0.06	0.04	0.09
	2	40	20/0.5		30	1.59	1.66	3.12	0.08	0.09	0.12
	3	50	30/0.5		40	3.95	2.01	5.96	0.13	0.14	0.14
	1	30	10/0.5	1	20	1.33	1.80	2.57	0.17	0.08	0.21
	2	40	20/0.5		30	2.07	2.17	5.83	0.33	0.19	0.35
	3	50	30/0.5		40	4.61	2.30	5.15	0.36	0.24	0.41
	1	30	10/0.5	1.4	20	1.41	2.29	3.07	0.21	0.17	0.22
	2	40	20/0.5		30	2.77	2.44	6.82	0.35	0.22	0.39
	3	50	30/0.5		40	4.82	3.48	8.23	0.41	0.32	0.48
*Mean*						*1.67*	*1.91*	*4.70*	*0.22*	*0.11*	*0.26*
*Sum*						*30.15*	*34.40*	*84.60*	*3.92*	*2.01*	*4.62*
*SD*						*1.42*	*0.60*	*1.94*	*0.13*	*0.09*	*0.14*
*Range*						*0.50*–*4.82*	*0.80*–*3.84*	*2.08*–*8.23*	*0.03*–*0.41*	*0.02*–*0.32*	*0.06*–*0.48*

The extracts were concentrated with a rotary evaporator, dried with the help of Nitrogen gas (N_2_), and weighed for extract yield and recovery. The final samples were dissolved in LCMS-grade EtOH, syringe-filtered (0.2 μm), and subjected to UHPLC-MSMS (1 ppb) for GZA quantification.

#### 2.4.2 US-MV (ultrasonciation method validation)

An in-house method was adopted [14], [15], [16] to evaluate and validate the US extraction efficiency with a slight change in the linearity range used. Twenty milligrams of the licorice sample was continually extracted with water until complete exhaustion of GZA from the sample (confirmed via UHPLC). The exhausted (blank) sample was divided into six parts and standard GZA solution was added to these samples in the linearity range of 1–800 ppb; sample-1 (1 ppb), sample-2 (50 ppb), sample-3 (100 ppb), sample-4 (200 ppb), sample-5 (400 ppb), and sample-5 (800 ppb). A single step US extraction was performed for all these samples, using the set of factors resulting in an optimum yield and recovery of GZA, based on US-MD results. The resultant extract was dried and processed for UHPLC-MSMS quantification as mentioned in US-MD.

### 2.5 UHPLC-MSMS MDMV

#### 2.5.1 UHPLC-MSMS MD

The GZA-standard stock solution was prepared in LCMS-grade EtOH (1 mg/mL), further diluted in order to prepare six working standard solutions in the linearity range of 1–800 ppb (1, 50, 100, 200, 400, 800) and filtered (0.22 µm). The liquid chromatography conditions for GZA method development consisted of a green mobile phase of H_2_O (A) and EtOH (B) using a gradient elution of 5–98% of B in a runtime of 10 min. This initial gradient was reduced to 5 min followed by 1 min after evaluation of various injection volumes for the sample (1, 5, and 10 μL) and flow rates of mobile phase (0.1, 0.2, and 0.3 mL/min) at column temperature of 40 °C. The mobile phase was free from any toxic solvents or additives.

The MS method for fragmentation of GZA at specific CE (collision energy) was optimized through precursor ion search followed by MRM (multiple reaction monitoring) for the specified daughter ions. The MS operating conditions were; ESI mode (-ve), collision gas (argon), drying and nebulizing gas (nitrogen), temperature for interface (300 °C) and desolvation line (250 °C), flow for drying and heating gas (10 L/min), and nebulizing gas (3 L/min), CID gas (270 kPa), nebulizer pressure (40 psi), scan time (1 min), scan rate (5/sec) whereas, the MS range to detect fragmentation was set at 100–1000 amu. The software used for data processing was Lab solution V 5.93 (Kyoto, Japan).

#### 2.5.2 Uhplc-MSMS MV

The chromatographic method developed for UHPLC-MSMS was validated according to the guidelines from International Council for Harmonisation (ICH) [17].

#### 2.5.3 Linearity

The coefficient of determination (r^2^-value) for GZA was constructed via the peak areas against samples concentrations used in the study. The linearity range was 1–800 ppb (1, 50, 100, 200, 400, 800 ppb) for GZA-standard drug.

#### 2.5.4 Precision

An inter- and intra-day precision for GZA was determined at three different levels of 100, 200, and 400 ppb of the standard drug. The data from triplicate run for each level was used to calculate mean and SD which were used to calculate the %RSD (relative standard deviation) for precision using the formula as shown below;

%RSD= (SD/mean)*100

#### 2.5.5 Accuracy/recovery

The accuracy for the method was determined at three levels of 80, 100, and 120%. The standard stock solution was spiked with the mentioned three levels of GZA-solution and triplicate readings were noted. The mean and SD for the data was processed and recovery for GZA was determined using the formula given below;

Recovery (%) = (b − a)/c × 100

a= (amount of drug in non-spiked samples), b= (amount of drug in spiked samples) whereas, c= (spiked amount added)

#### 2.5.6 Limit of detection (LOD)

A linear regression method and slope value of the regression equation was used to calculate the LOD for the method at a signal-to-noise ratio (S/N) of 3. The following formula was used to calculate the LOD.

LOD = 3.3*(SD of the intercept/slope)

#### 2.5.7 Limit of quantification (LOQ)

For LOQ, the linear regression method and slope vale with S/N = 10 were used. The equation for calculating LOQ is as below;

LOQ = 10*(SD of the intercept/slope)

### 2.6 US-UHPLCMSMS application in commercial samples

#### 2.6.1 US-application on commercial licorice samples

The US-MDMV (developed and validated method 2.4.1 & 2.4.2) was applied on commercially available samples of licorice in order to determine the discriminative power of the US technique for differentiation between samples from eight different geographical origins. For the practicality of the method on a large scale, 10 g of each sample was placed in 50 mL solvent and extracted individually, under the optimized set of factors (with maximum yield and GZA recovery). The liquid extract was filtered stepwise via cellulose filters (20 μm followed by 10 μm and 2 μm), rotary evaporated and dried via nitrogen stream. The dried sample was weighed for extract yield and %recovery, dissolved in LCMS grade EtOH, syringe-filtered (0.2 μm), and finally subjected to UHPLC-MSMS quantification.

#### 2.6.2 UHPLC-MSMS application in commercial licorice samples

The GZA amount was quantified in eight US-extracted licorice samples, using the developed and validated method of UHPLC-MSMS (2.5.1 & 2.5.2). The yields and SD for the GZA amount were calculated and determined.

## 3 Results

### 3.1 US-MD

#### 3.1.1 Extract yield (sum, mean, SD)

The general extract yield for the method was seen with a sum (mg/1mg) and mean (±SD) of 3.92 and 0.22 ± 0.13 (AC), 2.01 and 0.11 ± 0.09 (EtOH), 4.62 and 0.26 ± 0.14 for H_2_O. The range of extract yield in these solvents was 0.03–0.41 (AC), 0.02–0.32 (EtOH), and 0.06–0.48 for H_2_O (Table 1).

In terms of extract yield in individual solvents without the use of temperatures; samples 1–27 showed a sum (mg/1mg) and mean (±SD) of 1.82 and 0.20 ± 0.13 (AC), 0.52 and 0.06 ± 0.04 (EtOH), 2.21 and 0.21 ± 0.15 for H_2_O with a range of extract yield 0.03–0.38 (AC), 0.02–0.15 (EtOH), and 0.06–0.43 (H_2_O). For samples extracted in individual solvents under different set of temperatures (samples 28–54), a sum (mg/1mg) and mean (±SD) of 2.10 and 0.23 ± 0.13 (AC), 1.49 and 0.17 ± 0.09 (EtOH), 2.41 and 0.27 ± 0.14 (H_2_O) with an extract yield range of 0.06–0.41, 0.04–0.32, and 0.09–0.48 was observed for AC, EtOH, and H_2_O, respectively (Table 2). The order of extract yield in solvents was H_2_O > AC > EtOH whereas, for temperature (°C) 40 > 30 > 20 > 0, time (min) 3 > 2 > 1, pulse (seconds) 30/0.5 > 20/0.5 > 10/0.5, amplitude (%) 50 > 40 > 30, and particle size (mm) 1.4 > 1 > 0.5.

**Table 2:** Extract yields and GZA-amount recoveries in different individual solvents with and W/O the use of temperatures.

Sample #	US factors values tested				Temperature (°C)	Extract yield (mg/1mg) in different solvents			Solvent average yield and sum (SD)			Range
	Time (min)	Amplitude (%)	Pulse (seconds)	Particle size (mm)		AC	EtOH	H_2_O				
1–27	1	30	10/0.5	0.5	0	0.03	0.02	0.06	**AC**	Sum	1.82	0.03–0.38
	2	40	20/0.5			0.05	0.04	0.06		Mean	0.20	
	3	50	30/0.5			0.11	0.04	0.12		SD	0.13	
	1	30	10/0.5	1		0.10	0.03	0.19	**EtOH**	Sum	0.52	0.02–0.15
	2	40	20/0.5			0.27	0.05	0.26		Mean	0.06	
	3	50	30/0.5			0.34	0.07	0.41		SD	0.04	
	1	30	10/0.5	1.5		0.24	0.05	0.29	**H_2_O**	Sum	2.21	0.06–0.43
	2	40	20/0.5			0.30	0.07	0.39		Mean	0.25	
	3	50	30/0.5			0.38	0.15	0.43		SD	0.15	
28–54	1	30	10/0.5	0.5	20	0.06	0.04	0.09	**AC**	Sum	2.10	0.06–0.41
	2	40	20/0.5		30	0.08	0.09	0.12		Mean	0.23	
	3	50	30/0.5		40	0.13	0.14	0.14		SD	0.13	
	1	30	10/0.5	1	20	0.17	0.08	0.21	**EtOH**	Sum	1.49	0.04–0.32
	2	40	20/0.5		30	0.33	0.19	0.35		Mean	0.17	
	3	50	30/0.5		40	0.36	0.24	0.41		SD	0.09	
	1	30	10/0.5	1.5	20	0.21	0.17	0.22	**H_2_O**	Sum	2.41	0.09–0.48
	2	40	20/0.5		30	0.35	0.22	0.39		Mean	0.27	
	3	50	30/0.5		40	0.41	0.32	0.48		SD	0.14	

GZA-amount (μg/mg) in different solvents												
						**AC**	**EtOH**	**H_2_O**				
1–27	1	30	10/0.5	0.5	0	0.50	0.80	2.08	**AC**	Sum	6.26	0.50–0.87
	2	40	20/0.5			0.78	1.48	3.82		Mean	0.70	
	3	50	30/0.5			0.80	1.78	5.59		SD	0.25	
	1	30	10/0.5	1		0.78	1.11	2.53	**EtOH**	Sum	14.90	0.80–2.54
	2	40	20/0.5			0.80	1.72	4.86		Mean	1.66	
	3	50	30/0.5			0.85	2.00	5.63		SD	0.51	
	1	30	10/0.5	1.5		0.10	1.55	2.70	**H_2_O**	Sum	41.29	2.08–7.56
	2	40	20/0.5			0.79	1.93	6.51		Mean	4.59	
	3	50	30/0.5			0.87	2.54	7.56		SD	1.92	
28–54	1	30	10/0.5	0.5	20	1.33	1.35	2.56	**AC**	Sum	23.88	1.33–4.82
	2	40	20/0.5		30	1.59	1.66	3.12		Mean	2.65	
	3	50	30/0.5		40	3.95	2.01	5.96		SD	1.45	
	1	30	10/0.5	1	20	1.33	1.80	2.57	**EtOH**	Sum	19.50	1.35–3.48
	2	40	20/0.5		30	2.07	2.17	5.83		Mean	2.17	
	3	50	30/0.5		40	4.61	2.30	5.15		SD	0.61	
	1	30	10/0.5	1.5	20	1.41	2.29	3.07	**H_2_O**	Sum	43.31	2.56–8.23
	2	40	20/0.5		30	2.77	2.44	6.82		Mean	4.81	
	3	50	30/0.5		40	4.82	3.48	8.23		SD	2.07	

#### 3.1.2 GZA-amount (Sum, mean, SD)

The descriptive statistics for GZA amount (irrespective of temperature) revealed a sum and a mean (±SD) of 30.16 and 1.68 ± 1.42 (AC), 34.40 and 1.91 ± 0.60 (EtOH), 84.60 and 4.70 ± 1.94 μg/mg (H_2_O) whereas, the range for GZA (μg/mg) in each solvent was 0.50–4.82 (AC), 0.80–3.84 (EtOH), and 2.08–8.23 (H_2_O) (Table 1).

Samples 1–27 (extracted at ambient temperature), showed a sum and mean (±SD) of 6.28 and 0.70 ± 0.25 (AC), 14.90 and 1.6 ± 0.51 (EtOH), 41.29 and 4.59 ± 1.92 μg/mg (H_2_O) whereas, the range for GZA (μg/mg) in these solvents was 0.50–0.87 (AC), 0.80–2.54 (EtOH), and 2.08–7.56 (H_2_O).

Samples 28–54 (extracted at different temperatures), showed a sum and a mean (±SD) of 23.88 and 2.65 ± 1.45 (AC), 19.50 and 2.17 ± 0.61 (EtOH), 43.31 and 4.81 ± 2.07 μg/mg (H_2_O) whereas, the range for GZA in this set of samples was 1.33–4.82 (AC), 1.35–3.48 (EtOH), and 2.56–8.23 (H_2_O). Based on the sum and mean for 54 samples, both sets of extracts (with and without temperature) revealed a descending order for GZA amount as H_2_O > AC > EtOH. With respect to temperature, a drastic increase of GZA amount was observed with an increase of temperature from 20 → 40 °C. The highest yield of GZA (μg/g) further increased significantly with the use of temperature (ambient temperature → increased temperatures): AC (0.87 → 4.82), EtOH (2.54 → 3.84), H_2_O (7.56 → 8.23). The order for GZA yield at different temperatures was observed to be 40 °C > 30 °C > 20 °C > ambient temperature.

The increase in pulse (10 → 30 sec), amplitude (30 → 50 %), time (1 → 3 min), and particle size (0.5 → 1.4 mm) revealed a significant increase of GZA amount (μg/g), as seen in samples 1–27 (ambient temperature): AC (0.50 → 0.80) for 0.5 mm, (0.78 → 0.85) for 1 mm, (0.10 → 0.87) for 1.4 mm; EtOH (0.80 → 1.78) for 0.5 mm, (1.11 → 2.00) for 1 mm, (1.55 → 2.54) for 1.4 mm; H_2_O (2.08 → 5.59) for 0.5 mm, (2.53 → 5.63) for 1 mm, (2.70 → 7.56) for 1.4 mm. The descending order for GZA yield using these factors was: (pulse 30/0.5 sec, amplitude 50 %, time 3 min, particle size 1.4 mm) > (pulse 20/0.5 sec, amplitude 40 %, time 2 min, particle size 1.0 mm) > (pulse 10/0.5 sec, amplitude 30 %, time 1 min, particle size 0.5 mm).

The application of temperature with pulse (10 → 30 sec), amplitude (30 → 50 %), and time (1 → 3 min), further enhanced the GZA-amount (μg/g), evident from samples 28–54 (20 °C → 40 °C): AC (1.33 → 3.95) 0.5 mm, (1.33 → 4.61) 1 mm, (1.41 → 4.82) 1.4 mm; EtOH (1.35 → 2.01) 0.5 mm, (1.80 → 2.30) 1 mm, (2.29 → 3.48) 1.4 mm; H_2_O (2.56 → 5.96) 0.5 mm, (2.57 → 5.83) 1 mm, (3.07 → 8.23) 1.4 mm. The descending order for GZA-yield with application of temperature was: (pulse 30/0.5 sec, amplitude 50 %, time 3 min, particle size 1.4 mm) at 40 °C > (pulse 20/0.5 sec, amplitude 40 %, time 2 min, particle size 1.0 mm) at 30 °C > (pulse 10/0.5 sec, amplitude 30 %, time 1 min, particle size 0.5 mm) at 20 °C > (pulse 30/0.5 sec, amplitude 50 %, time 3 min, particle size 1.4 mm) at 0 °C.

For the selection of sample with more overall yield (optimum set-of-US-factors for extraction), a high individual GZA-amount of 8.23 μg/mg was noticed in H_2_O for 1.40 mm particle size, at 40 °C, time (3 min), pulse rate (30/0.5), and amplitude (50%), as shown in Table 2 whereas, a three dimensional re[resntation for the data obatianed from US-factors Vs extract yield and GZA-amount is shown in Fig. 2.

### 3.2 USMV (validation of optimum extraction-set of factors)

The validation of the method showed a high accuracy of 98.96% (±6.82) with r^2^ value = 0.995 in the linearity range of 1–800 ppb for spiked US-extracted samples. The LOD, LOQ, CC value, regression equation with slope and intercept value are presented in Table 3.

**Table 3:** MDMV parameters and data observed for US and UHPLCMSMS.

Parameters	MDMV values				
	**UHPLC-MSMS**	**US**			
Regression equation	y = 144.39x + 1121.1	y = 156.19x + 1082.2			
Slope	144.39	156.19			
Intercept	1121.1	1082.2			
Linearity range (ppb)	1–800	1–800			
Correlation coefficient (r^2^)	0.998	0.995			
SD of intercept	16.64	30.54			
LOD (ppb)	0.38	0.64			
LOQ (ppb)	1.15	1.95			
Accuracy	–	98.96 ± 6.82			
Accuracy and precision data for UHPLC-MSMS MDMV					
*Accuracy/%Recovery (*±*SD)*					
Low level (80%)	Medium level (100%)			High level (120%)	
98.63 ± 1.80	102.29 ± 0.63			101.45 ± 0.14	
*Precision (%RSD)*					
Inter-day			Intra-day		

Level 1	Level 2	Level 3	Level 1	Level 2	Level 3
1.23	0.53	0.42	0.26	0.17	0.13

### 3.3 UHPLC-MSMS MD

**Fig. 1:**
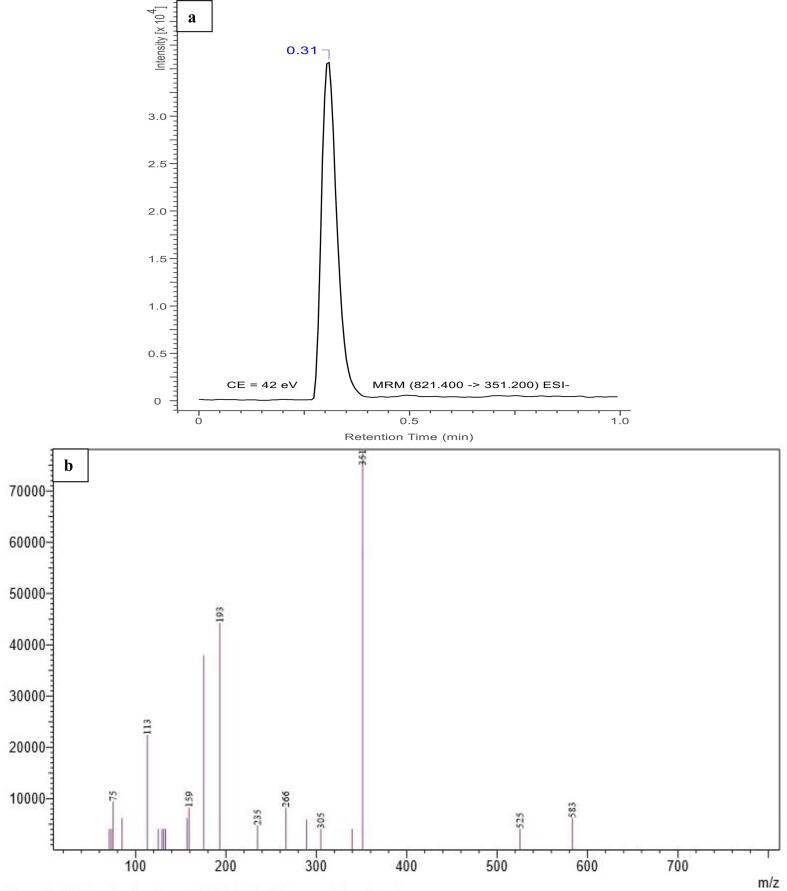
UHPLC chromatogram (RT = 0.31 min) a) MSMS fragmentation pattern for GZA (821.400 > 351.200) b).

The MS operating conditions developed for GZA consisted of –ve mode (ESI) fragmentation of precursor ion [M−H] ^-^ transition to daughter ions at (*m*/*z*) 821.40 → 351.20 → 193.25 → 113.10 with a base peak of 351.20 *m*/*z*. The CE optimized for the fragments were 42 (351.20), 46 (193.25), and 54 (113.10), dwell time (100.0 msec), event time (0.309), Q1 pre bias (V) of 28.0 (351.20, 193.25, and 113.10) whereas, Q3 pre bias (V) were 12.0 (351.20 and 113.10) and 23.0 (193.25). The acquisition time for MS was 1 min as shown in Fig. 1, with a proper fragmentation pattern for GZA.

The chromatographic conditions developed for UHPLC consisted of an isocratic mobile phase of A (H_2_O): B (EtOH) with composition 87:13, 1 min runtime, 0.3 mL/min flow rate for mobile phase, 3 μL injection volume, and 40 °C column temperature. The retention time (RT) for GZA was 0.31 min. For method development, two fragments of 351.20 and 193.25 *m*/*z* were selected whereas, for quantification and calibration *m*/*z* 351.20 was utilized based on its intensity and smooth peak shape. The GZA UHPLC-MSMS chromatogram (821.40 > 351.20 *m*/*z*) at RT of 0.31 min is shown in Fig. 1.

### 3.4 UHPLC-MSMS MV

The recoveries for the method were in the range of 99.21–100.16% with a mean recovery (±SD) of 100.81 ± 1.89 and RSD (1.88%). For precision, the %RSD was in the range of 0.42–1.23 (inter-day) and 0.13–0.17 (intra-day). The values for r^2^ = 0.998, LOD (0.38), and LOQ (1.15) showed reproducible results in the linearity range of 1–800 ppb. The details regarding the regression equation, intercept value, slope, levels used for accuracy, and precision are presented in Table 3.

### 3.5 Application of US-UHPLCMSMS in commercial samples (evaluation power of the method)

In order to determine the evaluation potential of the developed and validated US-UHPLCMSMS MDMV, eight samples from different origins were extracted and quantified for GZA-amount under optimized extraction set-of-factors; sample amount (10 g), particle size (1.4 mm), solvent (water), volume (50 mL), time (3 min), temperature (40 °C), pulse (30/0.5 sec), and amplitude (50 %). An average yield of 1.01 g/10 g with a % recovery of 10.11 was observed for the method. The sample with the highest yield and % recovery of 2.49 g/10 g (24.92 %) was the American licorice sample. For GZA amount, an average of 96.70 mg/10 g within a range of 71.28–125.84 mg/10 g was observed for the method in general. On the basis of individual GZA amount in eight licorice samples, the highest GZA amount (mg/10 g) was seen for Egyptian (125.84), followed by Pakistani (121.17), and Syrian licorice sample (118.76). The extract yield and GZA amount for all samples are shown in Table 4.

**Table 4:** The extract yield, recovery, and GZA-amount for 8 different geographical samples of licorice under using optimum set-of-factors.

No.	Origin	Sample amount (g)	Particle Size (mm)	Solvent (volume mL)	Time (min)	Temperature (°C)	Pulse (sec)	Amplitude (%)	Extract yield (g/10 g)	Recovery (%)	GZA-amount (mg/10 g)
1	Syria	10	1.4	H_2_O (50)	3	40	30/0.5	50	0.50	5.03	118.76
2	Egypt								0.32	3.16	125.84
3	America								2.49	24.92	77.73
4	Pakistan								0.64	6.42	121.17
5	India								0.77	7.69	99.44
6	Palestine								1.59	15.90	82.73
7	Georgia								0.64	6.38	76.67
8	Morocco								1.14	11.43	71.28
*Average yield*									*1.01*		
*Average %recovery*										*10.11*	
*Average GZA-amount*											*96.70*
*GZA-range*										*71.28*–*125.84*	

### 3.6 Statistical analysis

The data for US-MDMV and UHPLCMSMS-MDMV were categorized and analyzed using different statistical tests of General linear model of Univariate analysis (GLM-UniANOVA), Principal component analysis (PCA), Pearson’s correlation, K-mean cluster analysis (K-mean), and various relevant graphboard figures using SPSS software (Statistical Package the Social Sciences V 22.0).

#### 3.6.1 GLM-UniANOVA

The GLM-UniANOVA full factorial model with Bonferroni Post-Hoc Test, intercept model at *P = 0.05*, dependent variables (GZA-amount and extract yield), and fixed factors (solvent, temperature, time, pulse, amplitude, and particle size) were used to compare the main effects and confidence interval adjustment for the US-factors during US-MDMV and UHPLCMSMS-MDMV. The factorial models for GZA-amount Vs temperature*particle size*solvent (Fig. 3 a-c) and extract yield Vs temperature*particle size*solvent (Fig. 3 d-f) whereas, GZA-amount Vs time*pulse*amplitude (Fig. 4 a-c) and extract yield Vs time*pulse*amplitude (Fig. 4 d-f) were calculated for the *P-, F-* and *R-squared* values (Table 5). For both i.e. GZA-amount and extract yield the application with an increase in the temperature and particle size resulted in more yield in H_2_O. Likewise, GZA amount and extract yield were improved with an increase in temperature, amplitude, and pulse (*P ≤ 0.05*).

**Fig. 2:**
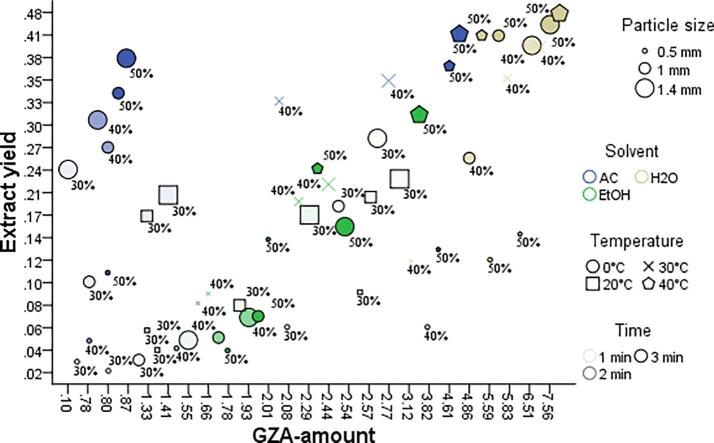
A comprehensive figure for the effect of US-factors (solvent*temperature*time*amplitude*pulse*particle size*time) Vs extract yield*GZA-amount.

**Fig. 3:**
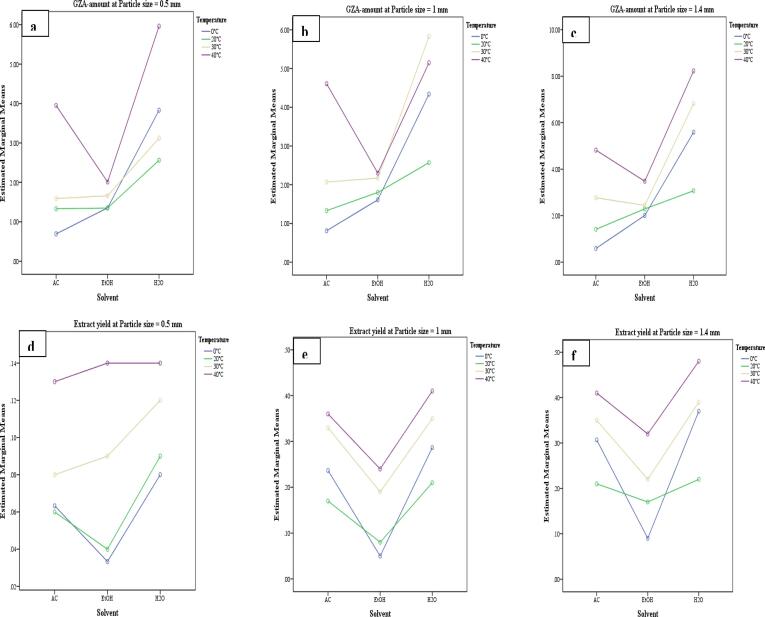
GLM-UniANOVA a-c) GZA-amount Vs temperature*particle size*solvent, d-f) Extract yield Vs temperature*particle size*solvent.

**Fig. 4:**
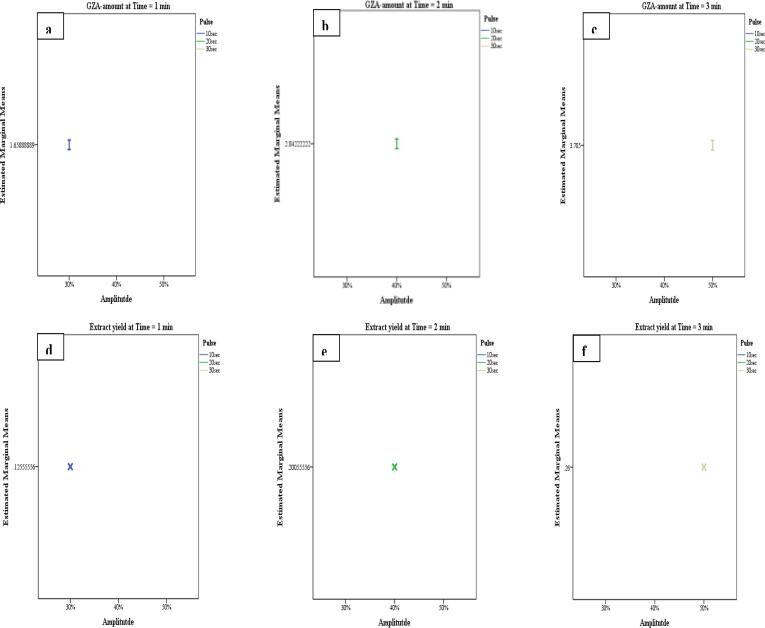
GLM-UniANOVA a-c) GZA-amount Vs time*pulse*amplitude, d-f) Extract yield Vs time*amplitude*pulse.

**Table 5:** the ANOVA table with *P-* and F- and R-values for PCA, and K-mean cluster analysis, and GLM-UniANOVA performed for the data obtained in USUHPLCMSMS-MDMV.

ANOVA table for K-mean cluster distribution and PCA					
Factors	F-value	Significance		Clusters	Samples
*K-mean cluster distribution for 54-samples US-MD and UHPLC-MSMS (US factors* Vs *GZA amount)*					
Z-score: Solvent	5.198	0.001		1	18
Extract yield	20.085	0.000		2	10
Z-score: GZA-amount	29.930	0.000		3	11
Z-score: Time	57.092	0.000		4	8
Z-score: Amplitude	57.092	0.000		5	7
Z-score: Pulse	57.092	0.000		***Total***	***54***
Z-score: Particle size	7.241	0.039			
Z-score: Temperature	14.618	0.000			

*K-mean cluster distribution for 8-samples US-UHPLCMSMS (Extract yield* Vs *GZA-amount)*					
Z-score: Geographical origin	5.842	0.061		1	3
Z-score: Extract yield	6.939	0.046		2	1
Z-score: GZA-amount	14.210	0.013		3	1
		4	3		
		***Total***	***8***		

*PCA for 54-samples US-MD and UHPLCMSMS (US-factors* Vs *GZA-amount)*					
Components	**PC1**	**PC2**	**PC3**		
Solvent	−0.075	−0.050	**0.952**		
Extract yield	0.387	**0.822**	0.224		
GZA-amount	0.416	0.326	**0.793**		
Time	**0.985**	0.051	0.042		
Amplitude	**0.985**	0.051	0.042		
Pulse	**0.985**	0.051	0.042		
Particle size	−0.088	**0.935**	−0.027		
Temperature	**0.465**	0.163	0.170		
*Individual %variance*	***43.288***	***21.174***	***20.236***		
*Cumulative %variance*	***43.288***	***64.462***	***84.697***		

*GLM-UniANOVA for 54-samples extracted with US-MD*					
Parameter	**Intercept model**	**F-value**	**P-value**	**R-squared value**	
GZA-amount	solvent*time*temperature	16.834	0.000	0.88	
	solvent *time*particle size	6.876	0.000	0.86	
	amplitude*pulse*time	6.300	0.004	0.198	
Extract yield	solvent*time*particle size	10.816	0.000	0.912	
	solvent*time*temperature	1.847	0.060	0.466	
	amplitude*pulse*time	5.318	0.008	0.173	

With regard to the main effects of US-factors upon GZA-amount and extract yield: the intercept models showed a more significant effect for solvent*GZA-amount as shown with high F-(16.834), R-squared (0.88), and significant *P-*value (0.000) whereas, for extract yield, more significant effect was observed from particle size i.e. extract yield*particle size revealed with high F-(10.816), R-squared (0.912), and *P*-value (0.000) (Table 5). The intercept models for GZA Vs solvent *time*particle size (F = 6.876, R-squared = 0.86, *P* = 0.000), and amplitude*pulse*time (F = 6.300, R-squared = 0.198, *P* = 0.004) whereas, for extract yield Vs amplitude*pulse*time (F = 5.318, R-squared = 0.173, *P* = 0.008) exhibited a significant effect however, the intercept model for extract yield Vs solvent*time*temperature (*P* = 0.06) was seen non-significant. This implicates the more correlation for solvent*GZA-amount rather than the solvent*extract yield alike particle size which skewed more towards extract yield (particle size*extract yield), compared to particle size*GZA-amount as discussed earlier with relevant values (*P ≤ 0.05*).

#### 3.6.2 PCA

**Fig. 5:**
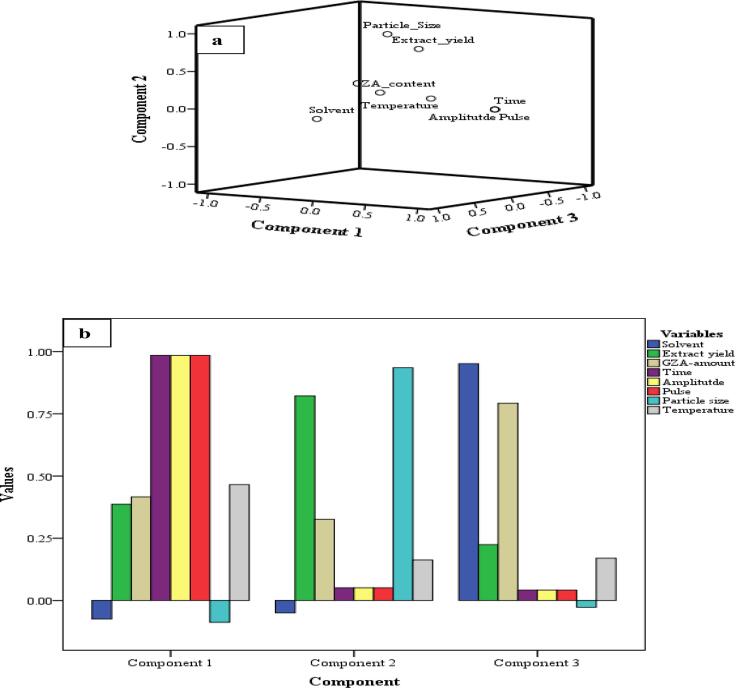
a) Three-dimensional presentation, and b) component loading for US-factors data.

An important statistical tool based on Eigenvalues (value approaching 1 shows more correlation), used to elucidate significant correlations among the variables of a dataset. The PCA for 54-samples of US/UHPLCMSMS-MDMV (GZA-amount, extract yield, solvent, temperature, time, particle size, pulse, amplitude) suggested distribution of data into 3-components (PC1-PC3) as shown in a three-dimensional scree plot along with a bar chart for factors distributed in the three components (Fig. 5a & b). The variability (%) for the three components is presented in Table 5. The cumulative variability for this dataset was 84.69% where PC1 (43.28%) represent the major individual variability followed by PC2 (21.17%), and PC3 (20.23%). A closer look for the distribution denotes the loading of time*amplitude*pulse*temperature in PC1 whereas, extract yield*particle size in PC2 and solvent*GZA-amount in a separate component of PC3. The data was significant at *P ≤ 0.05*. This implies a more significant correlation among time, amplitude, pulse, and temperature during extraction of GZA from licorice, as represented by the major %variability of the PCA. The remaining %variability is approximately equally shared by PC2 and PC3 where extract yield was loaded with particle size (PC2) and solvent with GZA-amount (*P ≤ 0.05*). This indicates a more significant correlation of extract yield with particle size whereas, GZA-amount is more correlated to the nature of solvent used. It is worthy to conclude; extract yield was more dependent upon particle size i.e. an increase in the particle size for licorice sample produced more extract yield whereas, GZA-amount was more prone to the nature of solvent i.e. solvent with more similarity for the structural and solubility index of intended extracting chemical is able to dissolve and extract more. Though the remaining factors revealed a significant general correlation, comparatively they were more inter-correlated rather than skewed towards a specific correlation as seen for solvent*GZA-amount and particle size*extract yield (*P ≤ 0.005*).

#### 3.6.3 Pearson’s correlation

A bivariate test used to determine the correlation among a set of variables which is based upon the scale of “0 (no correlation) → 1 (highly correlated) where a value of > 0.50 (irrespective of positive or negative)” is chosen to correlate the data point. The Pearson’s test was applied in this data in order to validate/verify the correlation pattern observed in PCA. It is evident further from Table 6 that particle size is more correlated with extract yield only whereas, GZA-amount with solvent alike PCA (*P ≤ 0.05*). The remaining factors studied (time, temperature, pulse, amplitude) were observed with a high correlation value = 1 in most of the instances but yet again this was a mere skewness towards a more inter-correlation (*P ≤ 0.05*).

**Table 6:** Pearson’s correlation for the USUHPLCMSMS-MDMV data with significant correlation.

	Solvent	Extract yield	GZA-amount	Time	Amplitude	Pulse	Particle size	Temperature	
Solvent		**1**							
Extract yield		0.120	**1**						
		0.388							
GZA-amount		**0.632**	**0.602**	**1**					
		**0.000**	**0.000**						
Time		0.000	**0.414**	**0.444**	**1**				
		1.000	**0.002**	**0.001**					
Amplitude		0.000	**0.414**	**0.444**	**1.000**	**1**			
		1.000	**0.002**	**0.001**	**0.000**				
Pulse		0.000	**0.414**	**0.444**	**1.000**	**1.000**	**1**		
		1.000	**0.002**	**0.001**	**0.000**	**0.000**			
Particle size		0.000	**0.625**	0.217	0.000	0.000	0.000	**1**	
		1.000	**0.000**	0.115	1.000	1.000	1.000		
Temperature		0.000	**0.343**	**0.386**	**0.354**	**0.354**	**0.354**	0.000	**1**
		1.000	**0.011**	**0.004**	**0.009**	**0.009**	**0.009**	1.000	

#### 3.6.4 K-mean with ANOVA (selection of optimum extraction set-of-factors)

**Fig. 6:**
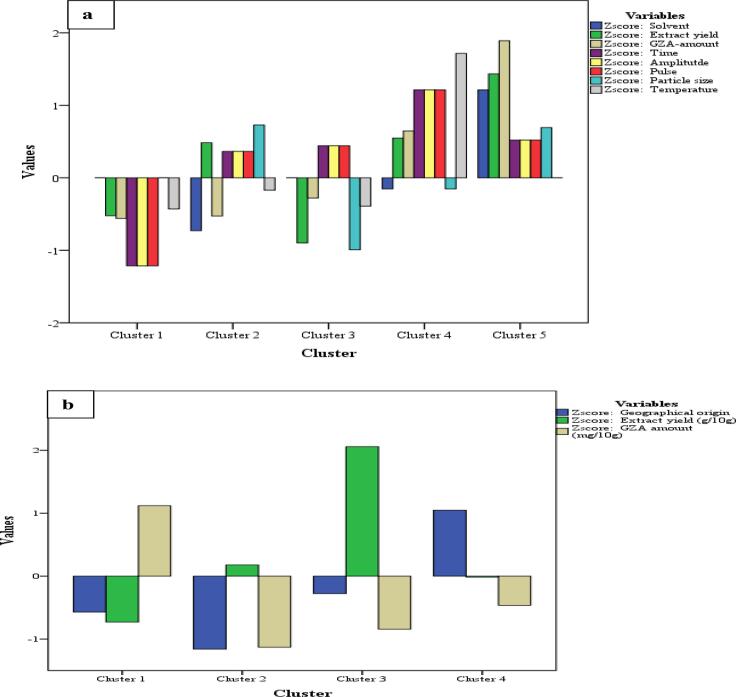
K-mean cluster distribution for a) USUHPLCMSMS-MDMV factors, and b) geographical samples studied for extract yield and GZA-amount.

A technique where a complex dataset is categorized into various clusters representing the observations with the nearest mean was applied to USUHPLCS-MD. This technique help select the set-of-factors resulting in higher extract yield and GZA amount in a pool of samples. For 54-samples of USUHPLCMSMS-MD, five clusters were suggested Cluster 1 (18 samples), Cluster 2 (10 samples), Cluster 3 (11 samples), Cluster 4 (8 samples), and Cluster 5 (7 samples). The details for the ANOVA table with F-value and significance level for each cluster is shown in Fig. 6a and Table 5. The pattern of correlations for these factors as discussed in previous sections was observed herein too. The details for clusters is as; Cluster 1 represents 18 samples with lack of correlation for any of the factors studied, Cluster 2 with 10 samples shows the correlation among all the factors except solvent*GZA-amount*temperature, Cluster 3 shows 11 samples where a high correlation was observed for time*amplitude*pulse only, Cluster 4 with 8 samples presents a lack of correlation for solvent*particle size with other factors whereas, Cluster 5 exhibited a strong intercorrelation for all the factors (solvent*extract yield*GZA-amount*time*amplitude*pulse*particle size*temperature) in 7 samples (*P ≤ 0.05*). These seven samples are: sample 41 (H_2_O, 2 min, 40%, 20 sec, 1 mm, 0 °C), sample 42 (H_2_O, 3 min, 50%, 3 sec, 1 mm, 0 °C), sample 44 (H_2_O, 2 min, 40%, 20 sec, 1.4 mm, 0 °C), sample 45 (H_2_O, 3 min, 50%, 30 sec, 1.4 mm, 0 °C), sample 50 (H_2_O, 2 min, 40%, 20 sec, 1 mm, 30 °C), sample 53 (H_2_O, 2 min, 40%, 20 sec, 1.4 mm, 30 °C), and sample 54 (H_2_O, 3 min, 50%, 30 sec, 1.4 mm, 40 °C) (*P ≤ 0.05*). Cluster 5 with seven samples representing the order of correlation is GZA-amount > extract yield > solvent > particle size > time = amplitude = pulse, suggests H_2_O as a solvent at the time (2 and 3 min), pulse (20 and 30 sec), amplitude (40 and 50 %), temperature (0, 30, and 40 °C), and particle size (1 and 1.4 mm) to be components for a set of factors with more yield and GZA-amount. With respect to high extract yield and more GZA-amount in these 7 samples, sample 54 was seen with a highest extract yield of 0.48 mg/1 mg and GZA-amount of 8.23 μg/mg. This suggests a set-of-factors for optimum GZA-extraction to be H_2_O (solvent) at 30 sec (pulse), 50% (amplitude), 3 min (time), 40 °C (temperature), and 1.4 mm (particle size) (*P ≤ 0.05*). A comprehensive figure for the multifactorial effect of US upon GZA-amount and extract yield is presented in Fig. 6a.

The K-mean cluster analysis was applied on samples from eight different origins analyzed as a part of the validation of the US-MDMV on a large scale. Four clusters were suggested for the 8 samples where Cluster 1 represent 3 samples (Egypt > Pakistan > Syria) with more GZA-amount only, Cluster 3 consists of 1 sample (American) with the highest extract yield only, Cluster 2 having 1 sample (Moroccan) with a medium level of both GZA-amount and extract yield, and Cluster 4 where the remaining 3 samples with less extract yield and GZA-amount were placed (Indian, Palestinian, and Georgian), as shown in Fig. 6b (*P ≤ 0.05*).

All the statistical tools were helpful to select the optimum set of factors for extraction and to successfully validate the US-MDMV for variation and evaluation of the quality of different origin licorice samples.

## 4 Discussion

This study investigated the multifactorial effects (solvent, time, temperature, particle size, pulse, amplitude) of US upon the extract yield and amount of natural sweetener GZA from licorice. Green solvents were used to develop (US-MD) a small scale optimum extraction method i.e. set of factors with more extract yield and GZA-amount, followed by validation US-MV (in-house method) and application of the US-MDMV on large scale for practical evaluation of the market available samples. To quantify GZA-amount in the extracted samples, a green UHPLCMSMS method was developed and validated according to ICH guidelines.

For US-MD, 1 mg of the sample was extracted using different levels of all the factors; solvent (AC, EtOH, H_2_O), temperature (0, 20, 30, 40 °C), pulse (10/0.5, 20/0.5, 30/0.5 sec), amplitude (30, 40, 50%), particle size (0.5, 1, 1.4 mm), and time (1, 2, 3 min) where 54-samples were extracted. The yield for each sample with the sum, mean (±SD), and range were calculated and analyzed statistically. The highest extract yield was obtained in 3 mins for the particle size of 1.4 mm in H_2_O at 40 °C using a pulse of 30/0.5 sec, and amplitude of 50% (finalized set-of-factors). These US-MD samples were analyzed for GZA amount with the help of an in-house developed UHPLC-MSMS method. A green, shorter (1 min run time), faster (RT for GZA = 0.31 min), and sensitive method (more accuracy and precision, r^2^ = 0.998) of GZA-quantification was developed and validated. Previously reported methods used both non-green solvents, longer run and retention time for GZA, or utilized comparatively low-sensitive techniques of HPLC/UV. (9; 10; 11; 12) The finalized set-of-factors were further validated and optimized using an in-house method (US-MV) [14], [16], [18] where blank samples were spiked with known concentrations of the standard GZA-drug in the linearity range of (1–800 ppb). The US-MV validated the set-of-factors for optimum extraction with an accuracy of 98.96 ± 6.82, r^2^ = 0.995, and LOD of 1.95 ppb. The USUHPLCMSMS-MDMV was finally applied at a large scale where 10 g of the sample amount from each licorice of different origins (eight samples collected at markets/malls in Khobar, Saudi Arabia) was used for extraction and GZA-quantification. A high extract yield with more GZA-amount was determined in these samples using K-mean cluster analysis and this method was successfully applied for practical evaluation/discrimination of the quality of food or herbal products available in the market for commercial use.

The statistical analysis of GLM-UniANOVA, PCA, K-mean, and Pearson’s correlation revealed a significant effect for (*P ≤ 0.05*) the studied factors during GZA extraction and quantification. More correlation was established for solvent*GZA-amount and particle size*extract yield. The remaining factors (time*temperature*amplitude*pulse rate) were more skewed towards intercorrelation (*P ≤ 0.05*). The K-mean cluster analysis opted the conditions used for samples with more extract yield i.e·H_2_O sample extracted at high temperature, pulse rate, amplitude, and particle size as an optimum set-of-US-factors for final extraction. The PCA followed by validation of Pearson’s correlation confirmed the significant correlation (*P ≤ 0.05*) for the studied factors in GZA-extraction however, the solvent*GZA amount as well as particle size*extract yield correaltion was comparatively more significant with high F-values as shown in Table 5 (supportive evidence and mechanism involved are discussed in forthcoming sections).

The distinctive features of the current US-UHPLCMSMS method are the use of green, non-toxic, eco- and human-friendly solvent i.e·H_2_O with least volume (10 mL in US-MD) and sample (1 mg in US-MD), extraction in lesser reported time (3 min) as compared to previous reports [9], [10], [1], [11], [13] with high yield at low temperature (40 °C), and high amount of GZA-quantified with the help of in-house greener, shorter, sensitive, and accurate analytical method. Additionally, it is a kind of cost- and time effective study where a complete dataset with mechanistic effects of multi-US-factors (Fig. 3c) Vs GZA-extraction and quantification from licorice (discussed below in detail) with a practical large-scale application for evaluation and quality determination of commercial licorice samples is reported for the first time.

### 4.1 Effect of solvent

**Fig. 7:**
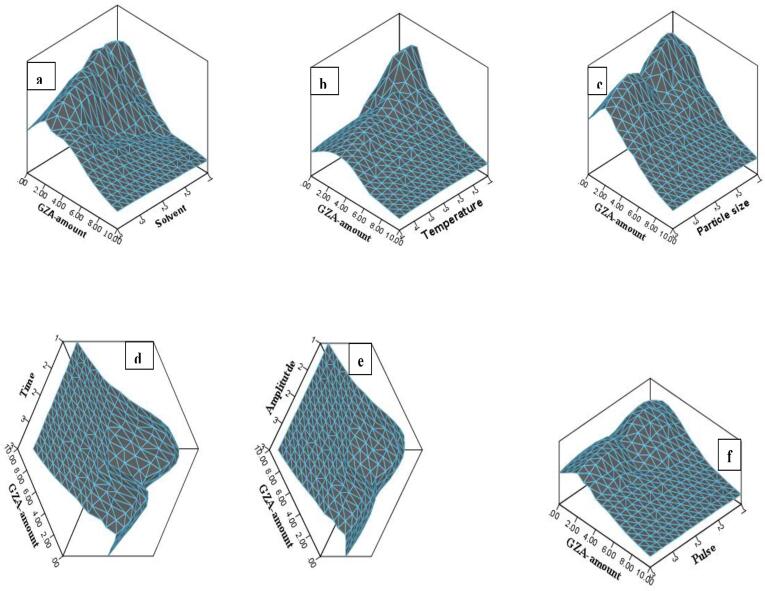
GZA-amount Vs a) solvent (1 = AC, 2 = EtOH, 3 = H_2_O), b) temperature (1 = 0, 2 = 20, 3 = 30, 4 = 40 °C), c) particle size (1 = 0.5, 2 = 1, 3 = 1.5 mm), d) time (1 = 1, 2 = 2, 3 = 3 min), e) amplitude (1 = 30, 2 = 40, 3 = 50%), and f) pulse (1 = 10/0.5, 2 = 20/0.5, 3 = 30/0.5 sec).

The selection of the nature of a solvent with alike structural, solubility, and polarity index for the extracting compound is utmost important during extraction i.e. polar solvents for polar compounds and vice versa. Furthermore, the use of green and non-toxic solvents is the need of the day [16]. Three different polarity solvents (AC, EtOH, H_2_O) were applied herein, where a high extract yield and GZA-amount was observed in H_2_O (Table 1 and Fig. 7a). Literature supports the use of H_2_O for more extract yield and GZA recovery [1]. Though a number of studies reported the use of binary mixtures (ethanol: water/methanol: water) in different ratios, herein methanol was excluded from the solvent list due to the green extraction process whereas, EtOH has been reported with a low recovery rate for GZA compared to methanol [11]. Similarly, the extraction medium with a higher viscosity has been reported with less mass transfer hence, a low extract yield and GZA-amount. Water owes the property of lower viscosity where more solubility with a high mass transfer for the extracting compound is achieved. A previous study reported the use of H_2_O in the extraction medium for higher GZA recovery [12]. It is more convenient to prepare/purify water in-house, using small distillation units or laboratory purifier which is a time- and cost-effective process. Keeping in mind these properties, H_2_O was used for GZA-extraction from licorice in this study.

*US-mechanism involved:* acoustic cavitation mechanism and threshold is widely affected by the viscosity and vapour pressure of the liquid. In order to initiate cavitation, the negative pressure of the rarefaction cycle may disrupt the cohesive forces of liquid molecule [19]. A solvent with a higher viscosity or surface tension and high vapour pressure increases the molecular interactions hence the threshold for cavitation. EtOH and AC carry a high vapour pressure of 30 and 12.4 kPa, respectively as compared to H_2_O (2.4 kPa) [20]. Water is a preferable medium for plant and food extraction as solvents with low viscosity, surface tensions, and vapour pressure carry more intense collapse of cavitation bubble [21].

### 4.2 Effect of temperature

For effective desorption and solubility of the compounds from a sample matrix, it is essential to cut down various interactions (diploe moments, hydrogen bonding, Vander Waals’s forces etc.) which are effectively achieved with a rise in temperature. This enhances the diffusion coefficient and mass transfer of the compounds into a solvent resulting in more yields [22]. This study used two sets of samples: 27 samples at different temperatures (20, 30, and 40 °C) and 27 samples at ambient temperature using the same parameters for extraction, in order to compare the effect of temperature upon the extract yield and GZA amount. An increase in extract yield and GZA-amount was observed with the use of temperature whereas; a dual counter effect was noticed with an increase in temperature up to 40 °C (Table 1 and Fig. 7b). Literature supports the use of 40 °C as an optimum extraction temperature for US applications, and a high extract yield for licorice/GZA was observed previously at 40 °C [12]. Furthermore, an increase in temperature beyond 40–50 °C is avoided in most of the plant or food extraction processes due to lack of extract yield improvement or decomposition of the sensitive compounds in the mixture as previously reported [1], [14], [23].

*US-mechanism involved:* food and pharmaceutical manufacturers prefer to use extraction and processing at the lowest possible temperature (20–40 °C) especially with US-related applications where sonochemical effects are produced at lower temperatures. This may be explained in the view of temperature effect Vs solvent viscosity. With an increase in temperature the viscosity of solvent decreases and vapours pressure increase. In the presence of high vapour pressure, more vapours enter the bubble cavity during cavitation process. This in turn weakens the violently collapsing property of the cavitation bubbles, ending up with a low/reduced sonochemical effect [24]. During the sonochemical process of acoustic cavitation, bubbles collapse and heat is produced in the local medium (5000 K) which may further increase the temperature of the solvent [25]. It is suggested to keep the temperature lower (below the boiling point of the solvent and destructive point of the targeted compound) for effective extraction processes where the nature of the targeted compounds remains preserved and intact.

### 4.3 Effect of particle size

Generally, an increase in particle size decreases the surface area and movement/transfer of molecules into the extraction medium and vice versa. A reverse phenomenon was observed herein i.e. more extract yield and GZA-amount was observed with an increase in particle size (0.5 → 1.4 mm), presented in Table 1 and Fig. 7c. A similar study was reported by Kumar, 2017 where optimum results were obtained for seed size >1[26]. Another study by Subroto et al., 2015 reported higher recovery for Jatropha whole kernel as compared to coarse and finely ground kernel powder [27]. The results for our study are in line with these reports.

*US-mechanism involved:* the cavitation bubble resulted during acoustic process collapses at the surface of solid–liquid interface with ultimate production of highspeed-liquid-jets (fast-moving stream of liquid) responsible for fragmentation or breakage of solid particles. The acoustic shockwaves and cavitation produce a macro-turbulence and interparticle mixing/collision which results in a reactivity with- and mass transfer into the liquid medium [28]. The mass transfer is aggravated with the formation of new surfaces exposed due to particle breakdown. The selection of an appropriate particle size is mandatory because the ineffectiveness of US-technique has been observed beyond a certain limit, particularly for particle sizes <1 mm. Albeit, further studies may clarify this concept in detail, the formation of void size is suggested to perturb the efficiency of US-technique in smaller particle size samples. The particle sizes with larger diameters may produce bigger voids hence, favour an ease-of-mass transfer for the compounds compared to smaller interparticle voids produced by smaller particle sizes with restricted mass transfer [27]. Due to decreased cavitational effect and smaller voids, the optimum particle size for US-technique may be >1.

### 4.4 Effect of time

In order to develop a faster and effective extraction, the extraction method was confined to the maximum possible short time (1 to 3 mins). With each incremental increase in time, more extract yield and GZA-amount was observed (Table 1 and Fig. 7d). Previous studies have reported an increase in GZA-amount with an increase in time. (10; 14; 28) It is essential to observe the effect of time Vs solvent, temperature, amplitude, and pulse. For more yield and GZA-amount, the decrease in time was compensated with an increase in amplitude and pulse as discussed in froth coming sections.

*US-mechanism involved:* selection of the most appropriate extraction time plays a vital role in cost-effectiveness and production of an optimum product. Verily, an increase in extraction time may result in more extract yield however, beyond a specific point the increase in time duration is no more effective as highlighted in the above-cited literature. With regard to US-technique, a sonocapillary effect is observed herein, where the liquid penetrates the pores of sample particles with the help of sonication and produces a swelling phenomenon (particularly in dry samples/powders) whereby an enlargement of cell wall pores is achieved. This swelling index with enlarging pore size may enhance with an increase in time under sonication, resulting in an increased extractive value via more desorption of the compounds to the external liquid medium [29]. According to the mass transfer concept, an increase in time may not be helpful beyond a certain point because the solvent saturation phenomenon is attained and the rate of mass transfer is decreased [13]. This necessitates either replacement with fresh solvent or selection of a more specific time for extraction.

### 4.5 Effect of amplitude

One of the driving force behind cavitation is amplitude i.e. the energy transmitted by amplitude is the source of violent collapse for cavitational bubble hence, an increase in amplitude is proportional to the formation of more violent bubble formation and sonochemical effect. This study used three different levels of amplitudes (30–50%) and an increase in extract yield and GZA-amount was observed with a linear increase of amplitude (Table 1 and Fig. 7e). The application of 50% amplitude with effective results was reported in a previous study which corroborates our results [26].

*US mechanism involved:* an increase in amplitude may increase the bubble formation during cavitation, producing jet-effect with an enhanced rupture or destruction of plant matrix. The more amplitude may transfer more energy to cavitational bubble to ensure violent rupture for jet-effect, yet again beyond a specific intensity the amplitude (similar to time duration) may cause the cavitational bubble to decrease. Since these bubbles need a delay during the rarefaction cycle (rarefaction-compression) in order to grow up to a required size for rupture, any unnecessary/abrupt increase in intensity, frequency, and amplitude energy may delay the bubble formation process with resultant less extract yield. For an effective US process, amplitude and intensity are more sensitive tools to be studied in-depth with practical results [30], [31]. Kumar, 2017 also reported a similar concept of an enhanced interfacial area contributing to more viscous film formation (area containing bubbles) when amplitude was increased [26].

### 4.6 Effect of pulse

Pulse and amplitude are the adjunct factors though, the availability of the pulse option in the US technique was utilized in this study at three levels (10/0.5, 10/0.5, and 30/0.5 sec). The increased pulse intensity enhanced the extract yield and GZA-amount (Table 1 and Fig. 7f). Alike amplitude, pulse rate has been studied and reported with comparatively effective results [28].

*US-mechanism involved:* the mechanism for pulse share the same concept of amplitude i.e. resonance is produced in the form of bubbles and lead towards the same cavitation phenomenon as discussed in detail in previous sections.

### 4.7 Effect of the geographical origin of samples

The developed and validated method of US extraction and UHPLCMSMS-quantification was successfully applied on licorice samples from eight different origins. The method was discriminative enough to evaluate the intra-samples variation of GZA-amount. Egyptian sample was found with more GZA yield whereas, the American sample produced a higher extract yield. Detailed information regarding the extract yield and GZA amount in these samples is presented in Table 4. This study does not aim to declare any sort of preference for one geographical origin over the other, on the contrary, it was performed to establish/validate large scale applications of the developed method for quality variation among food and herbal products available in the market. This variation in the quality is prone to various factors of water, salinity, altitude, humidity, temperature, collection, packing, storage, and shipment which needs to be studied further [15], [17].

## 5 Conclusion

A novel, green, and ecofriendly US method evaluating a set of various factors was developed for the extraction of GZA from licorice. US-MD resulted in a high extract yield and GZA-amount for licorice sample of particle size 1.4 mm within 3 min in H_2_O at 40 °C using amplitude and pulse of 50 % and 30/0.5 sec, respectively. The US-MD was further validated which exhibited high accuracy and r^2^ value showing the authenticity of the developed method. For quantification of GZA-amount, an in-house green, shorter (GZA-RT = 0.31 min), and reliable (validated for precision and accuracy with r^2^ = 0.998) UHPLCMSMS method was developed. The US-UHPLCMSMS method was applied in commercial licorice samples which further revealed the discrimination power of the method as evident from the complete characterization of the eight different geographical samples in terms of extract yield and GZA-amount. A set of the statistical tool was applied to the data obtained in USUHPLCMSMS-MDMV, where GLM-UniANOVA, PCA, K-mean, and Pearson's denoted a significant effect (*P ≤ 0.05*) for all the factors upon extract yield and GZA-amount i.e. with a linear increase in the level/value of the studied factor, an increase in extract yield and GZA-amount was observed. The mechanisms observed in US extraction consisted of cavitation, turbulence, sonocapillary, and sonochemical effect. This a novel and effective method for food and pharmaceutical manufacturers to extract and quantify GZA from licorice using a green process at low temperature with a cost- and time effective property.

## Funding source

The study did not receive any funding from any source.

## 7 Consent to publish

The consent for publication was obtained from all the authors.

## 8 Availability of data

The dataset used for analysis is completely mentioned and reported in the manuscript.

## Declaration of Competing Interest

The authors declare that they have no known competing financial interests or personal relationships that could have appeared to influence the work reported in this paper.
